# Description of an Improved Extension Apparatus for the Treatment of Fracture of the Thigh

**Published:** 1867-04

**Authors:** Gurdon Buck

**Affiliations:** Surgeon to the New York Hospital, St. Luke’s Hospital, etc., etc.


### Miscellaneous.

### Description of an Improved Extension Apparatus for the Treatment of Fracture of the Thigh.

INTRODUCED BY GURDON BUCK, M. D.,

*Surgeon to the New York Hospital, St. Luke's Hospital, etc., etc.*

*Articles Composing the Apparatus.*—Two bands of adhesive plaster spread on Canton flannel or thick twilled cotton; each band being two inches and a half wide and two feet long. At the end of one of the bands, a piece of elastic rubber webbing, two inches wide and ten long, is attached. At one end of the other band, a buckle of corresponding width, is fastened.

A thin block of wood three inches and a half wide transversely, and three inches vertically.

The perineal portion consists of rubber tubing of one inch calibre, having inside of it a tube of muslin stuffed with bran, and left an inch longer than the rubber tube at both ends. At each end of the muslin tube, a metalic ring is first fastened, and then shoved within the rubber tube, to the end of which it is also fastened. This arrangement preserves the rubber tube from being overstretched.

Two straps fastened to the rings at the ends of the perineal portion, serve to lengthen it and allow it to be made fast to the head of the bedstead.

A belt that passes around the opposite side of the body, and maintain the bearing of the perineal band in a line with the axis of the body and limb. The perineal portion should be wound with a narrow strip of Canton flannel or other soft material, and this should be changed as often as soiled.

Four guttered coaptation splints, covered with flannel, are intended to surround the fracture and be secured in place by three elastic bands, each having a buckle at one end.

An upright supporting a pulley wheel, to be fastened to the floor by three screws, opposite the foot of the bed.

*Mode of Application.*—The bands of adhesive plaster are first to be applied, one on either side of the limb from a point above the ankle upwards as high as the seat of fracture. The limb is then to be bandaged in the usual manner, beginning at the toes and covering the plasters, but leaving their lower ends free. The band of elastic webbing is next passed round the sole of the foot and fastened to the buckle on the other side of the foot. The block of wood should then be interposed between the loop of webbing and the foot. A cord fastened to the block thus adjusted is passed over the pulley, and has a weight suspended from it. This arrangement combines elasticity with the extending force, keeps the bands stretched out smooth, and prevents pressure upon the ankles. The amount of weight required must be proportioned to the resistance to be overcome, and the toleration of the patient. Sometimes five or six pounds only can be borne at the outset, and an increased weight subsequently.

After a fracture has taken place the sooner the limb is put up and subjected to treatment the better. Spasmodic twitchings of the muscles are controlled, and the patient made comfortable from the outset. To permit the application of lotions to the seat of injury during the first few days, the bandage should not be carried above the knee, and the ends of the plaster should be rolled up and kept in reserve. At the end of six or eight days the plasters may be extended up on the thigh, and the bandage continued over them. The coaptation splints are now to be applied around the thigh and secured by the three elastic bands. To complete the apparatus the perineal band should be adjusted and its ends fastened to the head of the bedstead, so as to be in a line with the axis of the body and limb. The limb should be raised on a hair cushion sufficiently to keep the heel from pressure.

In the employment of this method of treatment, experience has shown that in a large majority of cases the use of the perineal band may be dispensed with, the weight of the body being sufficient to resist the extending force. This resistance may be further increased by raising the foot of the bedstead five or six inches above the floor.

*Advantages of the Method.*—The advantages claimed for this method over others heretofore in use, are its great simplicity of arrangement, facility of management, and especially the comfort it affords the patient during a long confinement in bed. The efficiency with which uninterrupted extension of the limb can safely be kept up, secures, it is believed, better results than have been obtained by any other method. The sitting posture may be allowed without disturbing the action of the apparatus; an indulgence for which patients are always very grateful, and one which greatly alleviates the irksomeness of their condition. The materials required for employing this treatment are obtainable under almost any circumstances, the only indispensable article being adhesive plaster. If this is of the ordinary description, it is better to use it of double thickness. All the other articles requisite may be improvised. The elastic band may be dispensed with, and a round stick properly placed across the foot of the bedstead may serve instead of a pulley.

